# School feeding program has resulted in improved dietary diversity, nutritional status and class attendance of school children

**DOI:** 10.1186/s13052-018-0449-1

**Published:** 2018-01-23

**Authors:** Mastewal Zenebe, Samson Gebremedhin, Carol J. Henry, Nigatu Regassa

**Affiliations:** 1 School of Nutrition, Food Science and Technology, Hawassa University, P.O. Box: 05, Hawassa, Ethiopia; 2 School of Public and Environmental Health, Hawassa University, Hawassa, Ethiopia; 3 College of Pharmacy and Nutrition, 3302 Health Science E-wing, University of Saskatchewan, 104 Clinic Place, Saskatoon, SK S7N 2Z4 Canada

**Keywords:** School feeding program, Dietary diversity, Nutritional status, Southern Ethiopia

## Abstract

**Background:**

School Feeding Program (SFP) is a targeted safety net program designed to provide educational and health benefits to vulnerable children. However, limited evidence exists regarding the effect of the intervention on the nutritional status and school attendance of children. The study is aimed at examining the effects of SFP on dietary diversity, nutritional status and class attendance of school children in Boricha district, Southern Ethiopia.

**Methods:**

The study was conducted based on a representative data collected from 290 students drawn from the district. A school-based comparative cross-sectional study was conducted on school children aged 10–14 years. Data were collected using structured pretested questionnaire. The effects of SFP on dietary diversity score (DDS), class attendance rate, body-mass-index for age (BAZ) and height-for-age (HAZ) Z-scores were assessed using multivariable linear regression model.

**Results:**

The finding showed significantly higher mean (±SD) of DDS in SFP beneficiaries (5.8 ± 1.1) than the non-beneficiaries (3.5 ± 0.7) (*P* < 0.001). BAZ and HAZ of the beneficiaries were also higher than their counterparts, which were (0.07 ± 0.93), (− 0.50 ± 0.86) and (− 1.45 ± 1.38), (− 2.17 ± 1.15) respectively (P < 0.001). The mean (±SD) days of absence from school for non-beneficiaries (2.6 ± 1.6) was significantly higher than that of the beneficiaries (1.3 ± 1.7) (*P* < 0.05).

**Conclusion:**

Given the positive effects of the program in improving the DDS, nutritional status, and class attendance of school children, we strongly recommend scaling up the program to other food insecure areas.

## Background

Undernutrition contributes to about 8 million children death worldwide [1]. It is still a major public health problem in the developing countries, especially in the Sub-Saharan Africa [2]. Schools are uniquely positioned to promote healthy eating behaviors and attitudes among children; however, school-age children are not commonly included in health and nutrition surveys and an up-to-date overview of their nutritional status is not available [3].

In Africa, a substantial proportion of the school children suffer from malnutrition, are stunted, or experience short-term hunger [4]. Local studies in Ethiopia also indicated that undernutrition is a major public health problem. In 2015, about 31% of the school children were undernourished out of which 19.6% were stunted, 15.9% underweight and 14.0% wasted [5, 6].

Undernutrition in school children seriously affects their ability to learn [4]. The nutritional status of school-aged children impacts their health, cognition, and subsequently their educational achievement [7]. Poor health and inadequate nutrition among school-age children is likely to diminish their cognitive development either through physiological changes or by reducing their ability to participate in learning experiences - or both [8]. Hunger has also been a major barrier to child education. Accordingly, many school-age children in food insecure areas remain out of school [9].

According to the Food and Agriculture Organization of United Nation (FAO) [10], School Feeding Program (SFP) is a tool which enables children worldwide to attend school. In developed countries such as the USA, Japan, and the UK, millions of children benefit from SFPs that have been in place for years [10]. SFP provides benefits for disadvantaged children measured by indicators of physical growth and cognitive abilities [11]. In addition to reducing undernutrition, school feeding may also improve school enrollment, dropout and attendance [10].

However, the effects of SFP on nutritional and educational outcomes of school children remain debatable. Some studies have failed to witness the significant effect of SFP on class attendance rate [12]. While micronutrient deficiencies and hunger can be addressed through school feeding, the effect of SFP on children’s growth trajectory might be modest [13]. Studies have also indicated that school-age children may be too old to experience catch-up growth or recover from growth faltering [7].

Ethiopia’s national SFP is a joint program that involves the World Food Program (WFP) and the Federal Ministry of Education. In the study area the program was initiated in 2005. According to the program guideline, from Monday to Friday students receive a 150 g of hot lunch meal prepared either from wheat, corn or bean. In the country, despite the fact that SFP had been in place for a while, there is lack of adequate evidence regarding its effect on the nutritional status and school attendance of school children.

This study, therefore will contribute to gaining understanding of the effect of the program on the dietary diversity, nutritional status and class attendance of school children in chronically food insecure district of Boricha, Southern Ethiopia.

## Methods

### Study area

The study was conducted in Sidama Zone, Boricha district, Southern Ethiopia***,*** which is located 311kms south of Addis Ababa – the capital city of Ethiopia. The total number of primary schools in the district is 45. There are about 80,857 primary school children in the district, of which 42,306 are boys and 38,551 are girls. In 2016, in the district three primary schools were implementing the SFP and all of them were included in this study. The number of children attending school with the SFP is 5065–2673 boys and 2392 girls. According to the program standard, each SFP beneficiary student gets a 150 g of meal prepared from wheat, corn or bean once a day from Monday to Friday.

### Study design

School-based comparative cross-sectional study with both quantitative and qualitative components was conducted from January to February 2016. The quantitative study was done using both primary and secondary data. SFP beneficiary and non-beneficiary schools were matched based on pre-defined criteria.

### Study participants

All students aged from 10 to 14 years studying in schools with and without SFP in Boricha district were considered as the source population of the study; while, similar group of children enrolled in selected six schools of Boricha were taken as the study population.

All students registered in schools with feeding programs were considered as beneficiaries of the program; whereas, students from school not undertaking feeding program were considered as non-beneficiary. Children who were absent during the survey date were excluded from the study.

### Sample size determination and sampling technique

The sample size required for the study was calculated using Gpower software [version 3.1] considering; 95% confidence level, 90% power, 1:1 allocation ratio, 0.4 standardized mean difference effect size (equivalent to medium effect size) and 10% non response rate. The final sample size was 292 of which 146 were students from beneficiary schools, while another 146 were taken from non-beneficiary schools.

All the three schools that were implementing the SFP (Gesarakue, Hanjachafa and Shelo Abore) were included in the study. Among schools which were not implementing the feeding program, three schools were purposively selected using pre-defined matching criteria. The criteria were: (1) being nearest to the SFP implementing school, and (2) having a comparable kebele level (the smallest administrative unit in Ethiopia comprising approximately 1000 households) socio-demographic indicators (literacy rate and agro-ecological character). Accordingly, three SFP non-beneficiary schools (Mankite, Alawarfe and Sheloelancho) were included.

The sample size assigned to the two groups was distributed proportional to the size of the schools. Students were selected using a systematic random sampling technique by taking school rosters as a sampling frame.

### Data collection

In the study dietary diversity score (DDS), nutritional status (height-for-age z-score (HAZ) and body mass index (BMI)-for-age) and class attendance were the dependent variables while the SFP enrollment status was the exposure variable. Socio-demographic and economic factors including child’s age, maternal educational status, mother’s occupation, father’s occupation, household wealth index, size of agricultural land and household food insecurity status were considered as potential confounders.

Primary data were collected from the parents/primary caregivers of the index children by trained data collectors. Pre-tested structured questionnaire was used to assess social-demographic and economic characteristics, food security status, child characteristics and dietary diversity in both groups. The section of the questionnaire on socio-demographic characteristics was adopted from the standard Demographic and Health Survey (DHS) questionnaire.

The part of the questionnaire on DDS was adapted from the Food and Nutrition Technical Assistant (FANTA) guideline. The eight food groups DDS scale was used to assess the quality of diet based on foods consumed in the preceding day of the survey [14]. FAO’s 1 week Food Frequency Questionnaire (FFQ) having 16 food groups was used to compare the frequency of consumption of each food group between SFP beneficiary and non-beneficiary children [10].

FANTA’s Household Food Insecurity Access Scale (HFIAS) was used to assess the household food insecurity status of the households [14]. The scale explores the occurrence and frequency of occurrence of nine food insecurity related events in the past 30 days of the survey. HFIAS classifies the household as food secure, mild food insecurity, moderate food insecurity or severe food insecurity [14].

Household wealth status was assessed based on ownership of household assets (radio, television, chair, table, mobile phone, bicycle and horse or donkey cart), materials used to construct the house, numbers of livestock owned, ownership of improved drinking water source and latrine. Each household asset was assigned a score of zero or one, where an increased value indicates a better status; whereas, the number of livestock owned was entered as discrete numeric variable. Ultimately household wealth index was constructed using Principal Components Analysis (PCA) and the study participants were ranked into three tertiles; low, medium and high.

Anthropometric measurements were made by one of the investigators in order to avoid inter-observer variation. Measurements were made using calibrated equipments following standardized procedures**.** Body height and weight were measured with the child in light clothing and without shoes. The height of the children was measured to the nearest 0.1 cm using a measuring board, whereas weight was measured to the nearest 100 g using an electronic (Seca 770) scale. Ultimately, BMI-for-age and height-for-age (HFA) z-scores were computed based on the 2007 WHO growth reference data.

The qualitative data were collected through conducting key informant interviews with the concerned regional officer and focal person of the WFP and the principals of the six schools included in the quantitative study. Data were collected by one of the investigators using a semi-structured guide. All the interviews were tape recorded, translated and transcribed for analysis.

Secondary data were used to determine absenteeism and dropout rates of students. Absenteeism rate was determined as the number of days the child got absent from school in the preceding 2 weeks of the survey. School level dropout rate was determined for the academic year of 2015–16 for both beneficiary and non-beneficiary schools.

### Data management and analysis

The data were entered into EpiInfo [version 3.5.1] and then exported to SPSS [version 20] computer software program for statistical analysis. Descriptive statistics (frequency, percentage, measures of central tendency and dispersion) were used to summarize categorical and continuous variables.

Linear regression model was used to assess the net effects of the SFP on the dependent variables while controlling for potential confounders. Maternal education, mother’s occupation, husband’s occupation, agricultural land size, household food insecurity status and child age were found to be unbalanced variables in the groups being compared and hence controlled in the model. Assumptions of the model (normality and homoscedasticity of error terms, the absence of multicollinearity and linearity of association between the dependent and independent variables) were checked to be satisfied. The normality of the error terms was assessed using P-P plot and Kolmogorov-Smirnov test.

Anthropometric indices – HAZ and BAZ – were generated using WHO Anthro plus software [version2.0.2] based on the WHO 2007 growth reference data. Stunting and wasting were defined as Z-scores less than − 2 standard deviations.

For class attendance, the mean (±SD) of absence rate was calculated from the number of days the child got absent from school in the preceding 2 weeks and compared for both the beneficiary and non-beneficiary schools. The dropout rate obtained from the secondary sources were also compared between the groups.

The findings from the key informant interviews were translated, transcribed, and narrated according to coherent themes. The findings of the qualitative study were used to triangulate and complement the findings of the survey.

## Results

### Socio-demographic and economic characteristics

In this study 290 children – 145 from SFP-non beneficiary and 145 beneficiary schools – were included with the response rate of 99.3%. The majority of the respondents (89.0% in non-beneficiary schools and 76.6% among the beneficiary schools) were from Sidama ethnic group; while 11.0% and 22.1% of the respondents in the two groups respectively, were Wolyta in ethnicity. In both non-beneficiary and beneficiary groups protestant religion was the dominant group.

Almost all the respondent mothers were married and their mean age (±SD) was 34.6 ± 5.8 and 35.0 ± 6.7 years among beneficiary and non-beneficiaries, respectively. Most of the mothers, 62.1% among non-beneficiary and 82.8% of the beneficiary groups, did not attend formal education. Most of the fathers of the index children were farmers in both non-beneficiary (69.0%) and beneficiary (88.3%) schools. The mean (±SD) family size of the households was 7.3 ± 2.1 in non-beneficiary and 7.0 ± 1.5 in beneficiary groups. The mean (±SD) age of the children in non-beneficiary and beneficiary schools was 11.8 ± 1.1 and 12.4 ± 1.3, respectively.

Among the socio-demographic variables, the two groups were significantly different in maternal education, mother’s occupation, father’s occupation and age of the child (*P* < 0.05) (Table 1).

**Table 1:** Socio-demographic and economic characteristics of the school feeding program beneficiary and non-beneficiary children, Boricha district, Southern Ethiopia, Feb 3016

Variables	School feeding program		*p*-value^×^
	Non-beneficiaries (*n* = 145)	Beneficiaries (*n* = 145)	
Mean age (±SD) of the mothers	34.6 ± 5.8	35.0 ± 6.7	0.873
Mother’s educational status (%)			
No formal education	62.1	82.8	< 0.001*
Formal education	37.9	17.2	
Mother’s occupation (%)			
House wife	61.4	80.7	< 0.001*
Others	38.6	19.3	
Father’s educational status (%)			
No formal education	51.7	51.0	0.107
Formal education	48.3	41.0	
Father’s occupation (%)			
Farmer	69.0	88.3	< 0.001*
Others	31,0	11.7	
Mean(**±**SD) family size	7.3 ± 2.1	7.0 ± 1.5	0.069
Household wealth index (%)			
Lowest	36.6	31.7	0.041*
Middle	30.3	34.5	
Highest	33.1	33.8	
Mean(**±**SD) land size (hectare)	1.15 ± 1.79	0.66 ± 0.67	0.003*
Sex of the child (%)			
Male	60.0	61.4	0.999
Female	40.0	38.6	
Mean(**±**SD) age of the child (years)	11.8 ± 1.1	12.3 ± 1.3	0.021*

× Chi-square or independent t-test

* Statistically significant difference at *P* value of 0.05

There was a statistical significant variation on the wealth status of the non-beneficiary and beneficiary households. Among the beneficiaries, the proportion of households which fall under the lowest, medium and highest wealth tertiles were 31.7%, 34.5% and 33.8%; whereas, in the non-beneficiary households, the corresponding figures were 36.6%, 30.3% and 33.1%, respectively. All of the respondents in both groups owned plots of land for agricultural purpose. But the majority of the households, 78.6% in non-beneficiary and 91.7% in beneficiary households, had less than one hectare land.

In terms of latrine ownership, 86.9% of the non-beneficiary 98.6% of the beneficiary households had latrine. Among the non-beneficiary households 60.9% of them had access to improved water sources for drinking purpose; whereas, 33.8% of the households among the beneficiaries were using the same. Of the non-beneficiary respondents, 69.7% of them have used rudimentary materials to construct their home; whereas, 75.2% of the beneficiary respondents have used the same to construct their homes.

Among socioeconomic variables, agricultural land size and wealth index showed statistically significant difference between the two groups being compared (*p* < 0.05) (Table 1).

### Household food security status

Household food insecurity access scale was significantly different between the two groups (*p* = 0.008). A higher proportion of severe food insecurity was detected in non-beneficiary households (83.4%) than their counterparts (77.2%). Of the non-beneficiary households, 20.7% of them were enrolled in the productivity safety net program (PSNP) while 21.4% of the beneficiary households were part of the same program, but the difference was not statistically significant.

### Dietary diversity and SFP

Almost all children of non-beneficiaries (99.3%) and 93.8% of beneficiaries consumed grains and tubers in the preceding day of the survey. The pulse and legumes intake of the beneficiary households (91.7%) was substancially higher as compared with the non-beneficiaries (8.3%). There were also significant differences on the consumption of all other six food groups in favor of the beneficiary group (*p* < 0.05).

The mean (±SD) DDS among SFP non-beneficiaries (3.5 ± 0.7) was significantly lower than the beneficiaries (5.8 ± 1.1) (*p* < 0.001). The mean difference was 2.34 (95% CI: 2.12–2.55). In the linear regression model adjusted for potential confounders (child age, maternal educational status, family size, father’s occupation, household wealth index, size of agricultural land, and household food insecurity status); the DDS score was significantly higher among SFP beneficiaries (Table 2). The adjusted mean difference in DDS was 2.35 (2.10–2.60) in favor of the SFP beneficiaries.

**Table 2:** Association between enrollment in school feeding program and dietary diversity score among school children in Boricha district, Southern Ethiopia, February 2016

Simple linear regression			Multiple linear regression^a^		
*β* coefficient^b^	*t* statistic	*p* value	*β* coefficient^b^	*t* statistic	*p* value
2.34	21.532	< 0.001	2.35	19.058	< 0.001

^a^adjusted for child age, maternal educational status, mother’s occupation, father’s occupation, household wealth index, size of agricultural land and household food insecurity status

^b^coding scheme: 0 = non-beneficiaries, 1 = SFP beneficiaries

A one-week food frequency questionnaire having 16 food groups were used to compare the food group consumption of the beneficiary and non-beneficiary children. The frequencies of consumption for pulses and legumes; non-vitamin A rich vegetables; roots and tubers; and fats and oils were significantly higher for children from the SFP beneficiary group (*p* < 0.001).

### Nutritional status and SFP

The mean (±SD) HAZ score among school feeding program non-beneficiaries (− 2.17 ± 1.15) was significantly lower than the HAZ among beneficiaries (− 1.45 ± 1.38) (*P* < 0.001). The mean difference was 0.72 (95% CI: 0.43–1.01) in favor of the beneficiary children. In the regression model adjusted for child age, maternal educational status, mother’s occupation, family size, father’s occupation, household wealth index, size of agricultural land, household food insecurity status; the SFP beneficiaries showed better anthropometric status. The adjusted mean difference was 0.74 (95% CI: 0.42–1.06) (Table 3).

**Table 3:** Association between enrollment in school feeding program and anthropometric status of school children in Boricha district, Southern Ethiopia, February 2016

Outcome	Simple linear regression			Multiple linear regression^a^		
	*β* coefficient^b^	*t* statistic	*p* value	*β* coefficient^b^	*t* statistic	*p* value
Height-for-age z-score	0.720	4.819	< 0.001	0.735	4.533	< 0.001
BMI-for-age z-score	0.571	5.413	< 0.001	0.563	4. 807	< 0.001

^a^adjusted for child age, maternal educational status, mother’s occupation, father’s occupation, household wealth index, size of agricultural land, food insecurity status

^b^coding scheme: 0 = non-beneficiaries, 1 = SFP beneficiaries

The mean (±SD) of BAZ among the non-beneficiaries (− 0.50 ± 0.86) was significantly lower than the BMI among beneficiaries (0.07 ± 0.93) (*P* < 0.001). The net difference was 0.57(95% CI: 0.36–0.78). In the linear regression model adjusted for child age, maternal educational status, mother’s occupation, family size, father’s occupation, household wealth index, size of agricultural land, food insecurity status, the prevalence of stunting was higher in SFP non-beneficiaries (Table 3). The adjusted mean difference was 0.56 (95% CI: 0.33–0.79) in favor of the SFP beneficiaries.

### Class attendance and SFP

In this section, the number of days the child was absent from school was compared between the two groups. According to the mothers, absenteeism was reported more frequently among non-beneficiaries (91.0%) than beneficiary children (49.7%). The main reported reason for absence among non-beneficiaries was hunger (42.8%) while the leading reason in the other group was domestic workload (27.6%).

The mean (±SD) number of days a child absent from school in the preceding 2 weeks of the survey among SFP non-beneficiaries (2.6 ± 1.6) was significantly higher than the corresponding number of days for beneficiaries (1.3 ± 1.7) (*P* < 0.001). The mean difference was − 1.30 (95% CI: -1.68, − 0.91) days. In the linear regression model adjusted for potential confounders the mean difference was higher for SFP non-beneficiaries (Table 4). The adjusted mean difference was − 1.07(95% CI: -1.49,-0.65) days.

**Table 4:** Association between enrolment in school feeding program and absenteeism from class among school children in Boricha district, Southern Ethiopia, February 2016

Simple linear regressions			Multiple linear regression^a^		
*β* coefficient^b^	*t* statistic	*P* value	*β* coefficient^b^	*t* statistic	*P* value
−1.297	−6.650	< 0.001	−1.068	− 4.984	< 0.001

^a^adjusted for child age, maternal educational status, mother’s occupation, father’s occupation, size of agricultural land, food insecurity status

^b^coding scheme: 0 = non-beneficiaries, 1 = SFP beneficiaries

Secondary data on dropout rate was collected from the schools records for the year 2015/16 and compared between SFP beneficiary and non-beneficiary schools. The obtained result showed a slightly lower dropout rate for the beneficiary schools (0.9%) than the non-beneficiaries (1.7%).

### Findings from the qualitative study

All the school principals agreed on the positive effect of the program in alleviating short-term hunger in schools. They also explained that the feeding scheme has substancially improved the school enrollment, dropout and absenteeism problems of school-age children. Reportedly, the beneficiary students are pleased with the food provision and they do not experience hunger during school hours.

Finding from the key informant interview with the SFP regional officer and school principals reveal challenges of the program. Financial constrains frequently affect the timely and uninterrupted supply of grains and other inputs required for the program. Further, the agricultural unions responsible for supplying the grains frequently delayed delivery due to transportation problems. Shortage of potable water in the district and lack of clean grain storage site in the schools have also posed serious food hygiene concerns.

The other issue raised by the school principals was that educational time is being wasted while implementing the program and this could affect the educational quality of education.

## Discussion

The qualitative and quantitative studies have witnessed the positive effect of the SFP on the nutritional status and school attendance of school children in the district. The qualitative study has also identified key challenges of the program including financial constraints, delays in the delivery of supplies, food hygiene problems and wastage of academic time due to the feeding program.

The SFP appears to improve the dietary diversity of school children by adding different food groups into their diet [15]. The Boricha’s SFP also add at least two food groups on children’s daily diet so that the mean DDS of the beneficiary students was found higher than the non-beneficiaries. This result is consistent with the study done in Ghana on primary school children aged 7–16 years [16]. The study showed that the Ghana school feeding program has significantly improved the DDS of school children [16].

The findings from the current study also confirmed that the mean BMI-for-age z-score of the beneficiary students was significantly increased as compared with that of the non-beneficiaries. The finding is supported by a study from Bangladesh which reported increases in the BMI of school fed children compared to that of school children in the control group (17). Another study in Lao People’s Democratic Republic also support the current study showing that the SFP improved the nutritional status of school children by lowering the prevalence of wasting [7].

The mean HAZ of beneficiary students was higher than the non-beneficiary students which is similar with the study conducted in Lao People’s Democratic Republic that demonstrated a significant increase of HAZ in between-district with and without the SFP [7]. The finding from the qualitative study also supports the survey findings on the improvement of children’s nutritional status due to the SFP.

Our study witnessed a higher school attendance and lower percentage of dropout rates among SFP beneficiaries. This is consistent to a cross-sectional study done in Bangladesh, which showed boosted enrollment, increased attendance and reduced dropping out rate due to SFP [17]. Statistically significant difference was also observed on the reason for absence due to hunger among the non-beneficiaries and the beneficiaries [18]. On the contrary, a study done in Dale district, Southern Ethiopia found no significant positive impact of SFP on any of the three school participation indicators (enrollment, attendance and drop-out) **[**12]. This may be due to the fact that the data for the above study was collected between September and October which is a coffee harvesting season. In such seasons, school children might be expected to stay at home to support their family.

According to the data obtained from the key informant interview, the fact that the program could take much of the academic hours and take students away from their education may affect their academic performance. A parallel finding was reported by a study, which states the fact that the school feeding could take students educational time as a main disadvantage of SFP [11]. The key informant interview also identified some of the challenges of the SFP including storage problems and transportation resulting in delayed delivery of food which could be solved through more effective community participation.

The study should be interpreted inconsideration of the following limitations. Even though we have tried to control for potential confounders there may still be residual confounders from unmeasured variables. Error in reporting of child’s age and diets cannot be fully excluded. Furthermore, to assess dropout rate, report for only the year 2015/16 was used. This is because organized data for the previous years was not available for the non-beneficiary schools.

## Conclusion

The findings suggest that the SFP has improved the dietary diversity and nutritional status of school children. Further a higher percentage of attendance rates and lower dropout rate were observed among SFP beneficiary children.

Low community participation, storage and transportation problems were among the challenges faced during the implementation of the SFP and the fact that it took students’ educational time was raised as a major disadvantage of the program.

Given the positive effects of the program in improving the school children nutritional status, dietary diversity and class attendance, we strongly recommend scaling up the program in other food insecure areas. Finally, it is important to point out the need for a further longitudinal study which addresses the sustainability and potential long term impacts of the program for a better policy implication.
